# Uncertainty Analysis for Angle Calibrations Using Circle Closure

**DOI:** 10.6028/jres.103.008

**Published:** 1998-04-01

**Authors:** W. Tyler Estler

**Affiliations:** National Institute of Standards and Technology, Gaithersburg, MD 20899-0001

**Keywords:** angle metrology, autocollimators, calibration, closure, dimensional metrology, indexing tables, measurement uncertainty, optical polygons, self-calibration

## Abstract

We analyze two types of full-circle angle calibrations: a simple closure in which a single set of unknown angular segments is sequentially compared with an unknown reference angle, and a dual closure in which two divided circles are simultaneously calibrated by intercomparison. In each case, the constraint of circle closure provides auxiliary information that (1) enables a complete calibration process without reference to separately calibrated reference artifacts, and (2) serves to reduce measurement uncertainty. We derive closed-form expressions for the combined standard uncertainties of angle calibrations, following guidelines published by the International Organization for Standardization (ISO) and NIST. The analysis includes methods for the quantitative evaluation of the standard uncertainty of small angle measurement using electronic autocollimators, including the effects of calibration uncertainty and air turbulence.

## 1. Introduction

Full-circle calibrations of the angular divisions of polygon mirrors, indexing and rotary tables, and angle encoders can be accomplished without reference to separately calibrated reference artifacts using comparator techniques together with the principle of circle closure. The latter is a natural conservation law for plane angle, known since the time of Euclid, expressing the fact that the sum of the angles around any point in a plane equals 2*π* rad (360°). Should a circle be divided into *n* angular segments *A*_1_, *A*_2_, …, *A_n_*, and the difference between each segment and an unknown reference angle *X* be measured, closure provides a constraint on the data that enables a complete solution for all *n* + 1 unknowns. Circle closure is one of a number of self-proving comparator techniques employing multiple measurements together with suitable rearrangements of the components of a measurement system. A review of such techniques and their applications in dimensional metrology is given in Ref. [1].

In this paper we analyze two types of circle closure: a *simple closure*, just described, and a *dual closure* wherein two artifacts, such as a pair of indexing tables or an indexing table and an optical polygon, are inter-compared in such a way that each is calibrated in the process. The conceptual ideas here are well known and widely applied [2–9]; our major goal is to derive explicit expressions for the resulting measurement uncertainties in a manner consistent with guidelines published by the International Organization for Standardization (ISO) and NIST [10–11]. In the process, we show how closure constraints serve to reduce uncertainty in an interesting way as a result of a basic observation: the result of a measurement provides information not only about the measured quantity (i.e., the defined quantity subject to measurement, or the *measurand*), but also about any other quantities with which the measurand shares a functional relationship.

## 2. Simple Closure

In a simple closure calibration, the *n* angles of interest are independently compared to an unknown reference angle *X* using a difference measurement procedure. We consider the common case where the angles of interest *A*_1_, *A*_2_,…, *A_n_* and the comparison angle *X* are nominally equal to 2*π/n* radians. Denoting the deviations from the nominal value by *a*_1_, *a*_2_,…, *a_n_* and *x*, respectively, we have:

$$\begin{array}{l} A_{i}=2\pi /n+a_{i},i=1,2,\ldots ,n,\\ \;X=2\pi /n+x, \end{array}$$ (1)

where the deviations are assumed to be small angles that can be conveniently measured using an accurate autocollimator. With these definitions we have *A_i_* − *X* = *a_i_* − *x*, *i* = 1, 2,…, *n* and the constraint of circle closure becomes:

$$\begin{array}{l} 2\pi =\sum_{i=1}^{n}A_{i}=\sum_{i=1}^{n}\left(2\pi /n+a_{i}\right)\\ =n\cdot (2\pi /n)+\sum_{i=1}^{n}a_{i}=2\pi +\sum_{i=1}^{n}a_{i}, \end{array}$$

or, finally:

$$\sum_{i=1}^{n}a_{i}=0.$$ (2)

The physical realization of the reference angle *X* will depend on the chosen measurement procedure. Two particular procedures that lead to identical sets of measurement equations are (a) the calibration of an *n*-sided optical polygon using two autocollimators, and (b) the calibration of an indexing table, at a subset *n* of its discrete positions, using a dihedral mirror or a segment of an optical polygon. In the first case, *X* is the angle between the optical axes of the autocollimators; for the indexing table calibration, *X* is the angle between the two mirror normals.

**Fig. 1:**
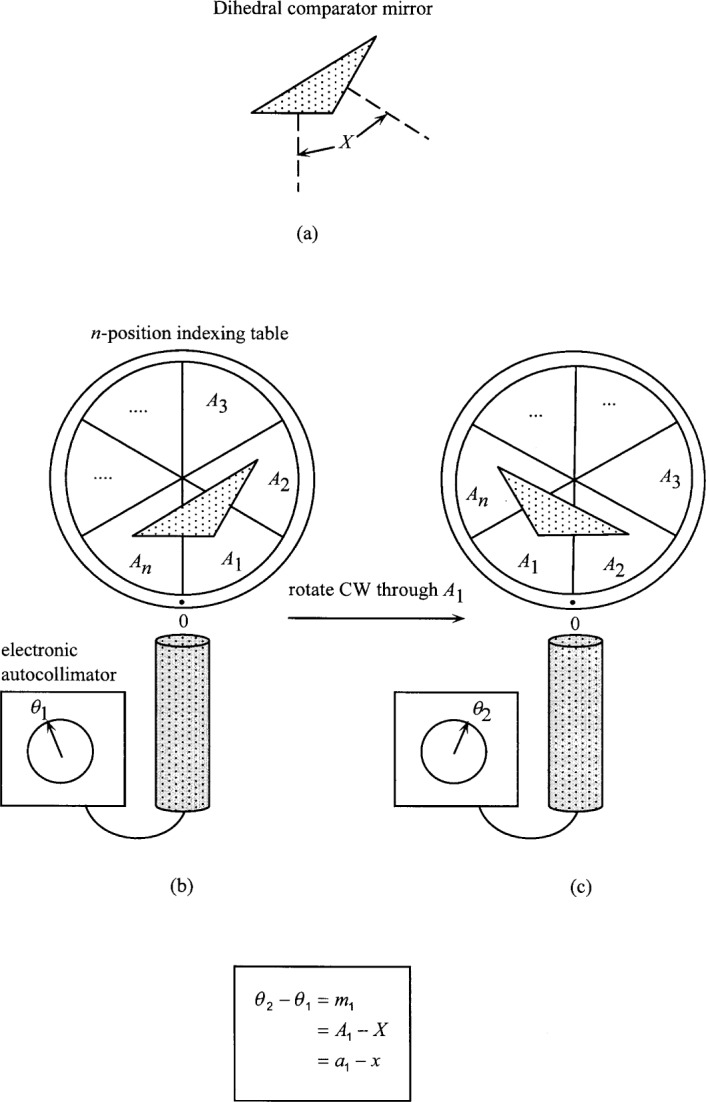
Calibration of an *n*-position indexing table using a dihedral comparator mirror and an autocollimator. The measurement sequence shown yields the first of *n* difference equations.

Figure 1 shows the setup for a simple closure calibration of an *n*-position indexing table using a dihedral comparator mirror (Fig. 1a) and an electronic autocollimator. The mirror is aligned so as to bring one of its faces near the null of the autocollimator, and a reading is taken (Fig. 1b). The table is then indexed through angle *A*_1_, rotating the second mirror face into near-normal incidence, and a second autocollimator reading taken (Fig. 1c). Denoting the measured angles by *θ*_1_ and *θ*_2_, we then have, for a suitable sign convention:

$$a_{1}-x=\theta_{2}-\theta_{1}=m_{1}.$$ (3)

where *m*_1_ ≡ *θ*_2_ − *θ*_1_. We then re-position the dihedral mirror to bring the first face back to a position near the autocollimator null and repeat the measurement sequence. Continuing in this fashion, we sequentially compare the angle *X* with each of the indexing table intervals, yielding the complete set of measurement equations:

$$\begin{array}{c} a_{1}-x=m_{1}\\ a_{2}-x=m_{2}\\ a_{3}-x=m_{3}\\ \vdots \\ a_{n}-x=m_{n}. \end{array}$$ (4)

Here we have *n* linear equations in the *n* + 1 unknown quantities {*a*_1_, *a*_2_,…, *a_n_*; *x*}, constrained by the closure relation, Eq. (2). Adding Eqs. (4) yields:

$$\sum_{i=1}^{n}a_{i}-nx=\sum_{i=1}^{n}m_{i},$$ (5)

and since the first sum vanishes we have the comparator angle deviation *x*, as an explicit function of the measurement data:

$$x=-\frac{1}{n}\sum_{i=1}^{n}m_{i}.$$ (6)

Now using Eq. (6) for *x* and the set of measurement relations Eq. (4), we have for the deviation of the *k*th segment of the indexing table:

$$\begin{array}{l} a_{k}=m_{k}+x\\ \;=m_{k}-\frac{1}{n}\sum_{i=1}^{n}m_{i}\\ \;=m_{k}-\frac{m_{k}}{n}-\frac{1}{n}\sum_{i\neq k}m_{i}\\ \;=\left(\frac{n-1}{n}\right)m_{k}-\frac{1}{n}\sum_{i\neq k}m_{i}. \end{array}$$ (7)

We note from the results, Eqs. (6) and (7), that the angular units for closure-based angle metrology are fixed by the scale of the small-angle measuring system, assumed here to be a calibrated autocollimator. The set of Eqs. (4) is invariant under the change of scale {*a_i_*, *x*, *m_i_*} → {*αa_i_*, *αx*, *αm_i_*}, *i* = 1, 2,…, *n* where *α* is an arbitrary scale factor. Thus, while closure obviates the need for separately calibrated comparator artifacts, the achievable uncertainty is limited by the degree to which the SI derived unit of plane angle (the radian) is realized by the small-angle measuring system.

## 3. Measurement Uncertainty

In order to evaluate the measurement uncertainty, we need the following result from the analysis of the propagation of uncertainty [10–11]. Suppose that a measur-and of interest *Y* is related to *n* independent and uncorrelated “influence quantities” *z_k_*, *k* = 1, 2,…, *n*, via the functional relation

$$Y=f(z_{1},z_{2},\ldots ,z_{n}).$$ (8)

In a procedure to determine *Y*, the quantities *z_k_* may be directly measured or simply estimated; in either case our state of knowledge of these quantities is described by a set of probability distributions with associated variances *u*^2^(*z_k_*). The positive square roots of these variances, *u*(*z_k_*), *k* = 1, 2,…, *n*, are called the *standard uncertainties* of the quantities *z_k_*. A first-order Taylor series approximation of *Y* = *f* (*z*_1_, *z*_2_,…, *z_n_*) then yields the estimated variance of *Y*:

$$u_{c}^{2}(Y)=\sum_{k=1}^{n}{\left(\frac{\partial f}{\partial z_{k}}\right)}^{2}u^{2}(z_{k}),$$ (9)

with the *combined standard uncertainty* of *Y* given by *u*_c_(*Y*). The quantities *∂f/∂z_k_* are known as *sensitivity coefficients* whose squares are essentially weighting factors for the individual variances *u*^2^(*z_k_*).

We emphasize here that the use of probability distributions to characterize our state of knowledge should not be taken to imply that the quantities *z_k_* are intrinsically random in nature. A dimensional measurement in engineering metrology is a deterministic exercise in which time and cost constraints determine the quantity and quality of the input measurement data. The quantities *z_k_* are generally a set of unknown constants whose values at the time the data were taken are not known exactly. Certain influences, such as the effect of air turbulence on the reading of an autocollimator, may appear at first sight to fluctuate randomly and unpredictably. When this happens we often choose to average over many readings in order to approximate a mean, or equilibrium, value for the measured angle. On the other hand, the time-dependent spatial distribution of the air density between an autocollimator and a target mirror could, in principle, be measured very accurately using interferometric techniques, and the result used to calculate, and correct for, the subsequent refraction error in the optical signal. That we normally choose not to do so is a matter of economics and not a decision forced on us because of any randomness inherent in the measurement process. In using a probability distribution to describe an experimental parameter, what is “distributed” is not the parameter itself but rather our knowledge with respect to the value it had when we performed the measurement.

For the comparator angle *x*, we then use Eq. (9) to compute the variance:

$$u_{c}^{2}(x)=\sum_{i=1}^{n}{\left(\frac{\partial x}{\partial m_{i}}\right)}^{2}u^{2}(m_{i}).$$ (10)

In Appendix A we show that for an accurately calibrated autocollimator measuring angular artifacts of high quality (in the sense that measured angles are reasonably small), the variance *u*^2^(*m_i_*) may be assumed to be constant for all measurements *m*_1_, *m*_2_,…, *m_n_*. We suggest two ways in which this constant value can be assigned in practice; for typical laboratory environments, it is reasonable to assume that:

$$u^{2}(m_{i})\approx \frac{2\beta^{2}}{N}s_{N}^{2}(\bar{R})\equiv u_{0}^{2}=\text{constant},$$ (11)

where *β* is a calibration parameter on the order of unity and $s_{N}^{2}(\bar{R})$ is the experimentally measured (Type A) variance of the mean $\bar{R}$ of a set {*R*_1_, *R*_2_,…, *R_N_*} of *N* autocollimator readings for a fixed target mirror position.

With this result, Eq. (10) becomes:

$$\begin{array}{c} u_{c}^{2}(x)=\sum_{i=1}^{n}{\left(\frac{\partial x}{\partial m_{i}}\right)}^{2}u_{0}^{2}\\ =\sum_{i=1}^{n}\left(\frac{1}{n^{2}}\right)u_{0}^{2}=n\cdot \frac{u_{0}^{2}}{n^{2}}=\frac{u_{0}^{2}}{n}, \end{array}$$ (12)

so that the combined standard uncertainty of *x* is:

$$u_{c}(x)=\frac{u_{0}}{\sqrt{n}}.$$ (13)

Equation (13) expresses what we might intuitively expect on the basis of repeated measurements in the presence of uncorrelated measurement “noise.” Since the unknown angle *x* has been the subject of *n* independent and uncorrelated difference measurements, the uncertainty of the measured value of *x* has been reduced by $\sqrt{n}$ from the uncertainty of a single measurement.

Then, using Eq. (10) and the assumed variances $u^{2}(m_{i})=u_{0}^{2}$, *i* = 1, 2,…, *n*, we have:

$$\begin{array}{l} u_{c}^{2}(a_{k})=\sum_{i=1}^{n}{\left(\frac{\partial a_{k}}{\partial m_{i}}\right)}^{2}u^{2}(m_{i})\\ ={\left(\frac{n-1}{n}\right)}^{2}u_{0}^{2}+\sum_{i\neq k}\left(\frac{1}{n^{2}}\right)u_{0}^{2}\\ =u_{0}^{2}\left[{\left(\frac{n-1}{n}\right)}^{2}+\frac{n-1}{n^{2}}\right]\\ =\frac{n-1}{n}u_{0}^{2}. \end{array}$$ (14)

Thus, for the combined standard uncertainty of *a_k_* we have:

$$u_{c}(a_{k})=\sqrt{\frac{n-1}{n}}\cdot u_{0},$$ (15)

with the same result for the remaining angular deviations. Equation (15) illustrates an interesting feature of calibrations that are constrained by subsidiary conditions such as circle closure. Given a single measurement of *a_k_*, we would expect an uncertainty equal to *u*_0_, whereas the actual result is less than *u*_0_ by a factor of $\sqrt{(n-1)/n}$. Each measurement of one of the deviations *a_i_* ≠ *a_k_* adds a little to our knowledge of *a_k_* because of the closure constraint. As the number of unknown deviations *n* increases, the factor $\sqrt{(n-1)/n}$ approaches unity and the reduction in uncertainty decreases, as we might expect. The closure constraint only provides a single piece of information whose effect is diluted as the number of unknowns becomes large.

## 4. Dual Closure

Indexing tables are commonly calibrated at a set of *n* positions using an intercomparison technique wherein the table of interest (the test table) is mounted concentrically atop a comparison table capable of indexing to the same *n* positions. Typically one table is rotated clockwise (CW) through a chosen angle, followed by a counter-clockwise (CCW) rotation of the second table through the same angle, so that a plane mirror target mounted on the top table is returned to a nominal zero position. Small differences in mirror position before and after indexing are measures of differences between pairs of nominally equal angular segments of the two indexing tables. These small difference angles are normally measured using an accurate autocollimator.

By the nature of the procedure, the angular positioning errors of both indexing tables are simultaneously determined by a complete set of measurement data. Therefore, in what follows, we no longer distinguish the “test table” from the “comparison table” but simply refer to them as the top table (T) and the bottom table (B), respectively. We denote the *n* angular segments of interest for the top and bottom tables, respectively, by *T_i_* = 2*π*/*n* + *t_i_* and *B_i_* = 2*π*/*n* + *b_i_*, *i* = 1, 2,…, *n*, where (*b_i_*, *t_i_*) are small angular errors. Then for all *i* we have *B_i_* – *T_i_* = *b_i_* – *t_i_*, and the circle closure constraints on the two tables can be written, by the steps leading to Eq. (2):

$$\begin{array}{l} \sum_{i=1}^{n}b_{i}=0\\ \sum_{k=1}^{n}t_{k}=0. \end{array}$$ (16)

## 5. Explicit Example: *n* = 3

**Fig. 2:**
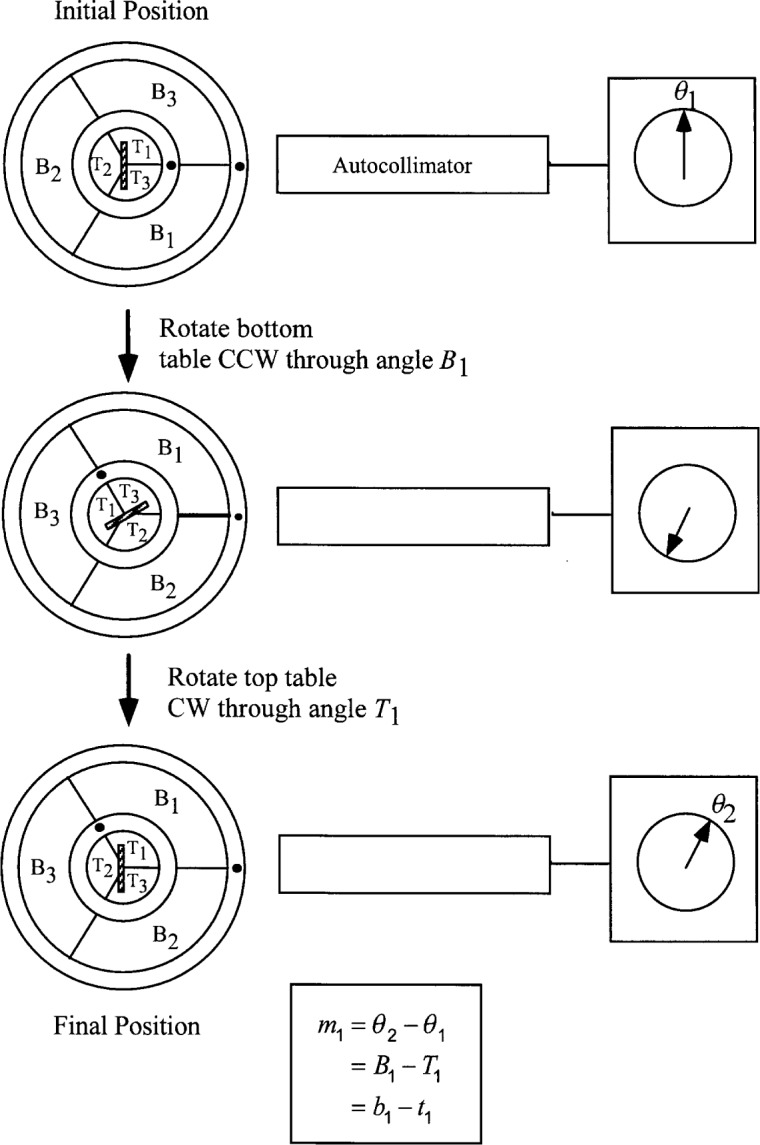
Dual closure calibration of a pair of 3-position indexing tables. The two-move measurement sequence shown yields the first of difference equations.

In order to demonstrate the calculation of measurement uncertainty for indexing table calibrations using dual closure, we will work out the results for two three-position tables in enough detail so that all steps in the procedure may be appreciated. We will then be in a position to generalize to the *n* × *n* case. We thus consider the setup shown in Fig. 2, with a pair of three-position tables.

We label the three segments of the top table {*T*_1_, *T*_2_, *T*_3_} and those of the bottom table {*B*_1_, *B*_2_, *B*_3_}, with segments *T*_1_ and *B*_1_ initially positioned as shown in the top diagram of Fig. 2. The small dots locate the zero index marks on each table. In acquiring measurement data, the bottom table always rotates CCW while the top table rotates CW, viewed from above.

Now consider the measurement sequence consisting of the following moves: (1) rotate the bottom table CCW through angle *B*_1_ and then (2) rotate the top table CW through angle *T*_1_. This sequence is illustrated in the middle and bottom diagrams of Fig. 2. The net result of the two moves is a CCW rotation of the bottom table through a nominal angle of *B*_1_ ≈ 2*π*/3 rad (120°) and a rotation of the target mirror through angle *B*_1_ – *T*_1_ = *b*_1_ – *t*_1_, for a suitable set of sign conventions. Denoting by *m*_1_ the difference between the initial and final autocollimator readings, we have

$$b_{1}-t_{1}=m_{1}.$$ (17)

We now repeat the measurement sequence two more times, yielding two more difference equations *m*_2_ = *b*_2_ – *t*_2_ and *m*_3_ = *b*_3_ – *t*_3_. At the end of the three measurements, the entire system is returned to its initial configuration. We then rotate the top table through angle *T*_1_ ≈ 120° CW, re-position the target mirror near the autocollimator null, and repeat the set of three two-move sequences. Proceeding in this manner yields a complete set of 3 × 3 = 9 difference measurements in which the three angles of the top table are each compared with the three angles of the bottom table. (We note here that this is only one of many different complete move sequences that yield 3 × 3 = 9 independent differences between pairs of table segments. For any given sequence, the nature and numbering of the coefficient matrix and data vector defined below will differ in detail from that derived here but the resulting uncertainty evaluations for the table angle deviations will be identical.)

The 3 × 3 indexing table difference equations resulting from a complete set of segment comparisons are given by

$$\begin{array}{l} b_{1}-t_{1}=m_{1}\\ b_{2}-t_{2}=m_{2}\\ b_{3}-t_{3}=m_{3}\\ b_{1}-t_{2}=m_{4}\\ b_{2}-t_{3}=m_{5}\\ b_{3}-t_{1}=m_{6}\\ b_{1}-t_{3}=m_{7}\\ b_{2}-t_{1}=m_{8}\\ b_{3}-t_{2}=m_{9}, \end{array}$$ (18)

where *m*_1_,…, *m*_9_ are differences between pairs of autocollimator readings. This over-determined set of nine linear relations among six unknown quantities has no unique solution. This can be seen by noting that set of equations is invariant under the additive transformations *b_i_* → *b_i_* + *K*, *t_i_* → *t_i_* + *K*, *i* = 1, 2,…, *n*, where *K* is an arbitrary constant. The equations are also not independent; we see, for example, that *m*_9_ = *m*_4_ + *m*_6_ − *m*_1_.

In order to proceed, we combine the measurement relations, Eqs. (18), with the two constraint Eqs. (16) and express the resultant set of linear equations in matrix form:

$$\begin{array}{cc} \left[\begin{array}{rrrrrr} 1 & 0 & 0 & -1 & 0 & 0\\ 0 & 1 & 0 & 0 & -1 & 0\\ 0 & 0 & 1 & 0 & 0 & -1\\ 1 & 0 & 0 & 0 & -1 & 0\\ 0 & 1 & 0 & 0 & 0 & -1\\ 0 & 0 & 1 & -1 & 0 & 0\\ 1 & 0 & 0 & 0 & 0 & -1\\ 0 & 1 & 0 & -1 & 0 & 0\\ 0 & 0 & 1 & 0 & -1 & 0\\ 1 & 1 & 1 & 0 & 0 & 0\\ 0 & 0 & 0 & 1 & 1 & 1 \end{array}\right] & \left[\begin{array}{c} b_{1}\\ b_{2}\\ b_{3}\\ t_{1}\\ t_{2}\\ t_{3} \end{array}\right]=\left[\begin{array}{c} m_{1}\\ m_{2}\\ m_{3}\\ m_{4}\\ m_{5}\\ m_{6}\\ m_{7}\\ m_{8}\\ m_{9}\\ 0\\ 0 \end{array}\right] \end{array}$$ (19)

or

Ax=m, (20)

where ***A*** is an 11 × 6 coefficient matrix, ***x*** is a 6 × 1 column vector of indexing table angular deviations: ***x*** = (*b*_1_*b*_2_*b*_3_*t*_1_*t*_2_*t*_3_) and ***m*** is an 11 × 1 vector of measurement data plus constraints. This is an over-determined set of 11 equations in 6 unknowns. Such data sets are in general *inconsistent* because of measurement errors, which means that there is no solution vector ***x*** which satisfies all eleven equations simultaneously. This is a standard problem in linear algebra and can be solved (in the sense of closest approximation) by the method of least squares. For a detailed development see, for example, Ref. [12]; in this paper we simply state the result.

The rank of a matrix is equal to the number of linearly independent rows (or columns) of the matrix. Given the set of linear equations ***Ax*** = ***m***, where the *m* × *n* matrix ***A*** has rank *n*, then the least-squares solution $\bar{x}=({\bar{b}}_{1}{\bar{b}}_{2}{\bar{b}}_{3}{\bar{t}}_{1}{\bar{t}}_{2}{\bar{t}}_{3})$is given by:

$$\bar{x}={\left(A^{⊤}A\right)}^{-1}A^{⊤}m,$$ (21)

where ***A***^T^ is the transpose of the coefficient matrix ***A***. In our 3 × 3 problem, it can be shown that the rank of ***A*** is equal to 6, as required, and so we proceed to work out the product matrix:

$$\begin{array}{l} {\left(A^{⊤}A^{⊤}\right)}^{-1}A^{t}=\frac{1}{27}\\ \times \left[\begin{array}{rrrrrrrrrrr} 7 & -2 & -2 & 7 & -2 & -2 & 7 & -2 & -2 & 6 & 3\\ -2 & 7 & -2 & -2 & 7 & -2 & -2 & 7 & -2 & 6 & 3\\ -2 & -2 & 7 & -2 & -2 & 7 & -2 & -2 & 7 & 6 & 3\\ -7 & 2 & 2 & 2 & 2 & -7 & 2 & -7 & 2 & 3 & 6\\ 2 & -7 & 2 & -7 & 2 & 2 & 2 & 2 & -7 & 3 & 6\\ 2 & 2 & -7 & 2 & -7 & 2 & -7 & 2 & 2 & 3 & 6 \end{array}\right] \end{array}$$ (22)

Then, using $\bar{x}$ and ***m*** above, with Eqs. (21) and (22), and carrying out the matrix multiplication, yields the least-squares estimates for the angle deviations of the indexing tables:

$${\bar{b}}_{1}=\frac{1}{27}[7(m_{1}+m_{4}+m_{7})-2(m_{2}+m_{3}+m_{5}+m_{6}+m_{8}+m_{9})],$$ (23)

$${\bar{b}}_{2}=\frac{1}{27}[7(m_{2}+m_{5}+m_{8})-2(m_{1}+m_{3}+m_{4}+m_{6}+m_{7}+m_{9})],$$ (24)

$${\bar{b}}_{3}=\frac{1}{27}[7(m_{3}+m_{6}+m_{9})-2(m_{1}+m_{2}+m_{4}+m_{5}+m_{7}+m_{8})],$$ (25)

$${\bar{t}}_{1}=\frac{1}{27}[-7(m_{1}+m_{6}+m_{8})+2(m_{2}+m_{3}+m_{4}+m_{5}+m_{7}+m_{9})],$$ (26)

$${\bar{t}}_{2}=\frac{1}{27}[-7(m_{2}+m_{4}+m_{9})+2(m_{1}+m_{3}+m_{5}+m_{6}+m_{7}+m_{8})],$$ (27)

$${\bar{t}}_{3}=\frac{1}{27}[-7(m_{3}+m_{5}+m_{7})+2(m_{1}+m_{2}+m_{4}+m_{6}+m_{8}+m_{9})],$$ (28)

Equations (23)–(28) display the explicit functional relationships between the indexing table angle deviations and the measurement data. The combined standard uncertainties can then be calculated by taking the appropriate derivatives according to Eq. (9). For the variance in the least-squares estimate of *b*_1_ we have, using Eq. (23)

$$u_{c}^{2}({\bar{b}}_{1})=\sum_{i=1}^{9}{\left(\frac{\partial {\bar{b}}_{1}}{\partial m_{i}}\right)}^{2}u^{2}(m_{i}).$$ (29)

As before, the quantities $\partial {\bar{b}}_{1}/\partial m_{i}$ are the sensitivity coefficients and here we see that measurements *m*_1_, *m*_4_, and *m*_7_ have greater weight, by a factor of 7/2, than that of the remaining measurements. This follows naturally since these three measurements depend directly on the value of *b*_1_ [see Eqs. (18)], while the remaining measurements depend on *b*_1_ only indirectly, by way of the constraint relations. As in the case of simple closure, the measurement data here consists of a set of independent autocollimator difference readings and we again make the reasonable assumption (see Appendix) that each measurement has the same uncertainty: $u^{2}(m_{i})=u_{0}^{2}$, *i* = 1, 2,…, 9. Then Eq. (29) becomes

$$u_{c}^{2}({\bar{b}}_{i})=\frac{1}{27^{2}}[3\times 7^{2}+6\times 2^{2}]u_{0}^{2}=\frac{19}{81}u_{0}^{2},$$ (30)

with the combined standard uncertainty given by

$$u_{c}({\bar{b}}_{1})=\frac{\sqrt{19}}{9}u_{0}.$$ (31)

The uncertainties of the remaining least-squares estimates $\left({\bar{b}}_{2},{\bar{b}}_{3},{\bar{t}}_{1},{\bar{t}}_{2},{\bar{t}}_{3}\right)$ are also given by Eq. (31).

## 6. Generalization for Two *n*-Position Indexing Tables

The procedure described for the 3 × 3 indexing table intercomparison may be generalized to the case of an *n* × *n* calibration. The set of all difference measurements *b_i_ – t_j_*, *i*, *j* = 1, 2,…, *n* between the angular segments of the two tables will yield a set of *n*^2^ equations analogous to Eqs. (18). Using the same angular segment labeling conventions, the general problem will have an angle deviation vector ***x*** with 2*n* components (*b*_1_ *b*_2_ …*b_n_ t*_1_ *t*_2_…*t_n_*), a coefficient matrix ***A*** of size (*n*^2^ + 2) × 2*n*, and a data vector ***m*** of length *n*^2^ + 2. Using a matrix manipulation code such as MATLAB or Mathematica^1^, it is not difficult to show by induction that ***A*** has rank 2*n* and that Eq. (23), for general *n*, becomes:

$$\begin{array}{c} {\bar{b}}_{1}=\frac{1}{3n^{2}}\cdot [(3n-2)(m_{1}+m_{n+1}\\ +m_{2n+1}+\ldots +m_{n(n-1)+1})\\ -2(m_{2}+m_{3}+\ldots +m_{n}+m_{n+2}+m_{n+3}+\ldots +m_{2n}\\ +m_{2n+2}+\ldots +m_{n^{2}})], \end{array}$$ (32)

with analogous expressions for ${\bar{b}}_{2},\ldots ,{\bar{b}}_{n}$ and ${\bar{t}}_{1},\ldots ,{\bar{t}}_{n}$. The numbering scheme makes it unwieldy to write these expressions explicitly, but the general rule is simply

$$\begin{array}{l} {\bar{b}}_{k}=\frac{1}{3n^{2}}[(3n-2)\times (\Sigma (\text{all}\;m’\text{s with explicit dependence on}\;b_{k}))\\ \;-2(\Sigma (\text{remaining}\;m’s))], \end{array}$$ (33)

and

$${\bar{t}}_{k}=\frac{1}{3n^{2}}[-(3n-2)(\Sigma (\text{all}\;m’\text{s with explicit dependence on}\;t_{k}))+2(\Sigma (\text{remaining}\;m’\text{s)})],$$ (34)

where *k* = 1, 2,…, *n.*

The calculation of the combined standard uncertainty proceeds in exactly the same way as that leading to Eq. (31), under the reasonable assumption (see Appendix A) that the combined standard uncertainty of each measurement *m_i_* is just $u_{c}^{2}(m_{i})=u_{0}^{2}.$ Examining Eq. (32), we see that the quantity multiplied by (3*n* – 2) contains *n* terms, while the quantity multiplied by 2 contains *n*(*n* – 1) terms. (The total number of *m*-terms is just *n*^2^, the number of measurements.) We can then write, using Eq. (29)

$$\begin{array}{l} u_{c}^{2}({\bar{b}}_{1})={\left(\frac{1}{3n^{2}}\right)}^{2}[n{\left(3n-2\right)}^{2}+4n(n-1)]u_{0}^{2}\\ \;=\frac{1}{9n^{2}}(9n-8)u_{0}^{2}\\ \;=\left(\frac{1}{n}-\frac{8}{9n^{2}}\right)u_{0}^{2}, \end{array}$$ (35)

so that the combined standard uncertainty is

$$u_{c}({\bar{b}}_{1})={\left(\frac{1}{n}-\frac{8}{9n^{2}}\right)}^{1/2}u_{0}.$$ (36)

The same result follows for the combined standard uncertainty of the remaining angle deviation estimates. Equation (36) is quite interesting. If we neglect the term in *n*^2^, then $u_{c}({\bar{b}}_{1})=u_{0}/\sqrt{n}$, which is what we might expect intuitively, since the unknown angle *B*_1_ has been independently compared with the *n* angles *T*_1_,…, *T_n_*. The factor 8/(9*n*^2^) reduces the uncertainty slightly as a result of the constraints due to circle closure. Since there are only two closure relationships for any *n*, while the number of measurements grows like *n*^2^, the reduction in combined standard uncertainty becomes insignificant for large *n*; for *n* = 12 for example, a common value, the correction is less than 5 %.

## 7. Conclusions

Angle calibrations using closure constraints are self-proving and can be accomplished without the need for separately calibrated reference artifacts or the services of a standards laboratory. The SI unit of angle is transferred by a small angle measuring system, typically a calibrated electronic autocollimator. The constraints lead to (usually modest) reductions in the combined standard uncertainties of the measured angles. These uncertainties can be readily calculated using the results derived in this paper. In common laboratory environments, the primary source of measurement uncertainty for a well-characterized autocollimator is turbulence noise on the optical signal. The minimum achievable uncertainty is limited by the degree to which such noise can be reduced.

## Footnotes

1 Certain commercial equipment, instruments, or materials are identified in this paper to foster understanding. Such identification does not imply recommendation or endorsement by the National Institute of Standards and Technology, nor does it imply that the materials or equipment identified are necessarily the best available for the purpose.

## 8. Appendix A. Autocollimator Measurement Uncertainty

An autocollimator is an optical instrument used to measure small rotations of a plane mirror target. The output (reading) of a typical electronic autocollimator is a number proportional to the angle between the normal to the target mirror and the instrument optical axis. Since there is no direct physical access to the optical axis, a single reading with a fixed target conveys no useful information. Autocollimators are therefore used in either a differential mode wherein the difference between two readings measures target mirror rotation, or in a nulling mode where changes in target orientation are sensed and used for optical system alignment or servo control.

A first-principles analysis of autocollimator measurement uncertainty is by its nature very complex. The measurand as defined is the angle between the normal to a perfectly flat mirror and the propagation vector of a perfectly plane wavefront emitted by, and subsequently collected and focused by, a perfect optical system onto a perfectly linear detector. In practice, all of these features are realized only approximately. We are saved by the fact that real autocollimators, while useless for absolute measurements of angular position, can be accurately calibrated to measure small rotations or *changes* in angular orientation, even with imperfect optics and target mirrors that are not really flat.

The response of an autocollimator is in general nonlinear, especially for large angles near the limits of the measuring range. For calibrations typical of those considered in this work, the setup can be arranged so that all measurements occur near the center of the instrument range so that the response can be assumed to be linear. Calibration data for a particular instrument will serve to justify this assumption. For a single reading *R* of such an autocollimator, the sensed angle *θ* is given by:

θ=βR (A1)

where *β* is a proportionality constant derived from the instrument calibration. Because of refraction caused by turbulence in the air path between the autocollimator output aperture and the target mirror, the instrument reading will fluctuate about a mean value. It is common measurement practice to minimize this air path, employing shielding where possible, and to reduce the effects of turbulence by averaging over many readings.

Now suppose that a small rotation angle is measured by averaging *N* autocollimator readings at each of two mirror positions (*θ*_1_, initial, and *θ*_2_, final) and then taking the difference of these averages. Denote the two sets of readings by *R*_1_*_j_* and *R*_2_*_k_*, *j*, *k* = 1, 2,…, *N*. Using Eq. (A1), we then have for the measured angles:

$$\begin{array}{l} {\bar{\theta }}_{1}=\frac{\beta }{N}\sum_{j=1}^{N}R_{1j}=\beta {\bar{R}}_{1},\\ {\bar{\theta }}_{2}=\frac{\beta }{N}\sum_{k=1}^{N}R_{2k}=\beta {\bar{R}}_{2}, \end{array}$$ (A2)

and the measured mirror rotation *m* is just

$$\begin{array}{c} m={\bar{\theta }}_{2}-{\bar{\theta }}_{1}=\frac{\beta }{N}\sum_{k=1}^{N}R_{2k}-\frac{\beta }{N}\sum_{j=1}^{N}R_{1j}\\ =m(\beta ,R_{21},R_{22},\ldots ,R_{2N};R_{11},R_{12},\ldots ,R_{1N}), \end{array}$$ (A3)

where the last expression shows the explicit dependence of *m* on the measurement data. We then compute the variance of *m*, using Eq. (9):

$$\begin{array}{c} u_{c}^{2}(m)={\left(\frac{\partial m}{\partial \beta }\right)}^{2}u^{2}(\beta )\\ +\sum_{k=1}^{N}{\left(\frac{\partial m}{\partial R_{2k}}\right)}^{2}u^{2}(R_{2k})\\ +\sum_{j=1}^{N}{\left(\frac{\partial m}{\partial R_{1j}}\right)}^{2}u^{2}(R_{1j}). \end{array}$$ (A4)

From Eq. (A3), the sensitivity coefficients are given by:

$$\frac{\partial m}{\partial \beta }=\frac{1}{N}\sum_{k=1}^{N}R_{2k}-\frac{1}{N}\sum_{j=1}^{N}R_{1j}={\bar{R}}_{2}-{\bar{R}}_{1},$$ (A5)

and

$$\frac{\partial m}{\partial R_{2k}}=\frac{\partial m}{\partial R_{1j}}=\frac{\text{β}}{N}.$$ (A6)

The variance *u*^2^(*β*) in Eq. (A4) characterizes the standard uncertainty associated with the autocollimator calibration; for the variances *u*^2^(*R*_2_*_k_*) and *u^2^*(*R*_1_*_j_*) we use the Type A (statistical) evaluation estimates (sample variances):

$$\begin{array}{c} u^{2}(R_{2k})=s^{2}(R_{2k})=\frac{1}{N-1}\sum_{i=1}^{N}{\left(R_{2i}-{\bar{R}}_{2}\right)}^{2},\\ u^{2}(R_{1j})=s^{2}(R_{1j})=\frac{1}{N-1}\sum_{i=1}^{N}{\left(R_{1i}-{\bar{R}}_{1}\right)}^{2}, \end{array}$$ (A7)

With the results Eqs. (A5)–(A7), Eq. (A4) becomes

$$\begin{array}{l} u_{c}^{2}(m)={\left({\bar{R}}_{2}-{\bar{R}}_{1}\right)}^{2}u^{2}(\beta )+\frac{\beta^{2}}{N^{2}}[s^{2}(R_{2k})+s^{2}(R_{1j})]\\ ={\left({\bar{R}}_{2}-{\bar{R}}_{1}\right)}^{2}u^{2}(\beta )+\frac{\beta^{2}}{N}[s^{2}({\bar{R}}_{2})+s^{2}({\bar{R}}_{1})], \end{array}$$ (A8)

where $s^{2}({\bar{R}}_{2})=s^{2}(R_{2k})/N$, the experimental variance of the mean ${\bar{R}}_{2}$ and similarly for $s^{2}({\bar{R}}_{1})$. For a particular environment, the measurement noise induced by turbulence is normally stable in the sense that the standard deviation of the mean of a sample of *N* readings is sensibly constant from sample to sample. We therefore assume that $s^{2}({\bar{R}}_{2})=s^{2}({\bar{R}}_{1})\equiv s_{N}^{2}(\bar{R})$ = a constant for each sample of *N* readings. We then have for the variance of the angle measurement *m*

$$\begin{array}{c} u_{c}^{2}(m)={\left({\bar{R}}_{2}-{\bar{R}}_{1}\right)}^{2}u^{2}(\beta )+\frac{2\beta^{2}}{N}s_{N}^{2}(\bar{R})\\ =m^{2}\frac{u^{2}(\beta )}{\beta^{2}}+\frac{2\beta^{2}}{N}s_{N}^{2}(\bar{R}), \end{array}$$ (A9)

where we have used Eq. (A3) in the form ${\left({\bar{R}}_{2}-{\bar{R}}_{1}\right)}^{2}=m^{2}/\beta^{2}$. Equation (A9) is the central result for an autocollimator measurement with a negligible linearity error. The first term on the right-hand side arises from calibration uncertainty, as measured by the square of the relative standard uncertainty *u*(*β*)/|*β*|. The second term, arising from turbulence noise in the autocollimator signal, can be reduced by increasing the number of autocollimator samples *N* at the cost of increased measuring time and susceptibility to thermal drift.

The dependence of $u_{c}^{2}(m)$ on *m*^2^ means that each measurement in a full-circle angle calibration will, in general, have a different combined standard uncertainty. In practice, the set of individual variances can be approximated by a single constant value with negligible increase in overall measurement uncertainty. There are two ways to effect this approximation.

We first note that for an accurately calibrated autocollimator and typical high-quality angular artifacts, the measurement uncertainty will often be dominated by turbulence noise. To see this, we examine Eq. (A9) using a representative set of measurement parameters. A modern electronic autocollimator is adjusted by the manufacturer to set *β* ≈ 1, and after calibration we could expect a worst-case relative standard uncertainty of *u*(*β*)/|*β*| ≤ 0.1 % = 10^−3^. In the process of calibrating an indexing table with a dihedral mirror matched to the nominal angles of interest, the maximum angle that we might expect to measure would be on the order of 25 μrad (1 μrad ≈ 0.2″). Thus *m*^2^*u*^2^(*β*)/*β*^2^≤ 6.3 × 10^−4^ μrad^2^. In a typical measurement setup at NIST, with an air path of several centimeters between the auto-collimator and the target mirror, we average over several hundred autocollimator readings per measuring position and commonly measure turbulence noise values near $s_{N}^{2}(\bar{R})/N\approx 0.25{\mu \text{rad}}^{2}$.

Comparing these numbers, we see that in such experimental conditions the measurement uncertainty due to turbulence noise is larger than that due to calibration error by more than two orders of magnitude. Thus we can, with negligible error, set $u_{c}^{2}(m)\approx (2\beta^{2}/N)s_{N}^{2}(\bar{R})\equiv u_{0}^{2}$, a constant for all measurements, with the actual value assigned to $u_{0}^{2}$ determined by locally measured turbulence noise characteristics.

A second, more conservative way of assigning a constant uncertainty is to use the maximum value computed from Eq. (A9), based on the measurement data. Setting *m*_MAX_ = MAX{*m*_1_, *m*_2_,…*m_n_*}, we then fix a constant value for $u_{0}^{2}$ by setting

$$u_{0}^{2}=u_{c}^{2}(m_{\text{MAX}})=m_{\text{MAX}}^{2}\frac{u^{2}(\beta )}{\beta^{2}}+\frac{2\beta^{2}}{N}s_{N}^{2}(\bar{R}).$$ (A10)

This will result in overestimating the variances and combined standard uncertainties of some of the measured angles, but the turbulence component will still account for most of the uncertainty in practice. Either of the techniques just described can be used for quantitative uncertainty estimation. It is only necessary to give a complete description of the estimation procedure and any relevant approximations.
